# Retrospective derivation and validation of a search algorithm to identify extubation failure in the intensive care unit

**DOI:** 10.1186/1471-2253-14-41

**Published:** 2014-05-23

**Authors:** Muhammad Adeel Rishi, Rahul Kashyap, Gregory Wilson, Sara Hocker

**Affiliations:** 1 Multidisciplinary Epidemiological and Translational Research in Critical Care Medicine (METRIC), Mayo Clinic, Rochester, MN, USA; 2 Division of Critical Care Neurology, Mayo Clinic, Rochester, MN, USA

**Keywords:** Extubation failure, Search algorithm, Derivation, Validation, ICU

## Abstract

**Background:**

Development and validation of automated electronic medical record (EMR) search strategies is important in identifying extubation failure in the intensive care unit (ICU). We developed and validated an automated search algorithm (strategy) for extubation failure in critically ill patients.

**Methods:**

The EMR search algorithm was created through sequential steps with keywords applied to an institutional EMR database. The search strategy was derived retrospectively through secondary analysis of a 100-patient subset from the 978 patient cohort admitted to a neurological ICU from January 1, 2002, through December 31, 2011(derivation subset). It was, then, validated against an additional 100-patient subset (validation subset). Sensitivity, specificity, negative and positive predictive values of the automated search algorithm were compared with a manual medical record review (the reference standard) for data extraction of extubation failure.

**Results:**

In the derivation subset of 100 random patients, the initial automated electronic search strategy achieved a sensitivity of 85% (95% CI, 56%-97%) and a specificity of 95% (95% CI, 87%-98%). With refinements in the search algorithm, the final sensitivity was 93% (95% CI, 64%-99%) and specificity increased to 100% (95% CI, 95%-100%) in this subset. In validation of the algorithm through a separate 100 random patient subset, the reported sensitivity and specificity were 94% (95% CI, 69%-99%) and 98% (95% CI, 92%-99%) respectively.

**Conclusions:**

Use of electronic search algorithms allows for correct extraction of extubation failure in the ICU, with high degrees of sensitivity and specificity. Such search algorithms are a reliable alternative to manual chart review for identification of extubation failure.

## Background

In the intensive care unit (ICU) patient population, extubation failure is generally defined as requiring endotracheal reintubation within 72 hours of prior extubation [1]. The negative consequences of extubation failure include increased duration of mechanical ventilation, increased ICU length of stay (LOS), increased nosocomial pneumonia, and increased mortality [2,3].

The prevalence of extubation failure ranges from 2 to 25% depending on the population studied and the time frame (24–72 h) included for analysis [4]. In spite of its high prevalence in the ICU, extubation failure remains difficult to predict [5]. This dilemma stems partly from a lack of rigorous scientific investigation and partly from obstacles in defining the time period when a reintubation took place in the critically ill patient – information that usually is buried within the medical record. This is further compounded by inadequate search strategies or improper data filtering from the electronic medical record (EMR). Therefore, in order to address this problem, two elements must be defined: the reintubation episode and the time took place, in reference to prior extubation.

Recently, automated search strategies have been used to identify certain elements in a patient’s EMR [6,7]. Recently, Smischney et al. [7] developed and validated automated electronic search strategies to identify emergent intubations from EMRs. These investigators found that by using the electronic search strategies, they were able to achieve a sensitivity and specificity that were greater than 95%.

The primary objective of the present study was to develop and validate an automated electronic search strategy for extubation failure in the ICU. Identifying reintubation is a necessary first step before establishing its consequences and predictors. Our secondary aim was to compare the sensitivity, specificity, and positive and negative predictive values of our electronic search strategy with a comprehensive manual review of the medical record (the reference standard), from the EMR to detect extubation failure in patients on mechanical ventilation for a period of time in the ICU.

## Methods

### *Study population*

The derivation and validation subsets were obtained retrospectively from neuroscience ICU (NICU) at Mayo Clinic in Rochester, Minnesota. These subsets were a heterogeneous population of NICU patients admitted from January 1, 2002, through December 31, 2011. Although patients in other locations, such as the post anesthesia care unit (PACU), may have extubation failure, they are potentially different in regard to their pathophysiology. Therefore, the present study focused on population of critically ill patients.

**Figure 1:**
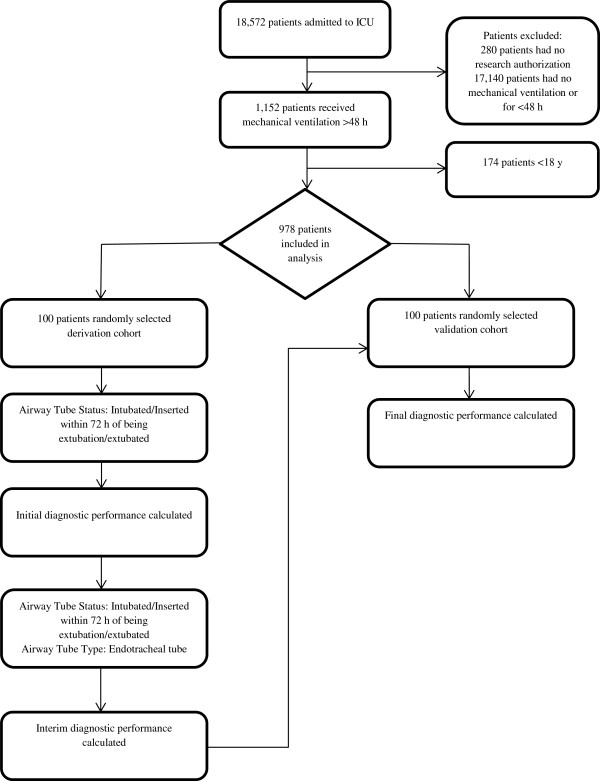
**Electronic search strategy flow diagram of patients in the Mayo Clinic life Science System from January 2002 through December 2011.** ICU indicates intensive care unit.

During the study period, 18,572 consecutive patients records admitted to the NICU were reviewed (Figure 1). A final cohort of 978 patients, ≥ 18 years of age and on mechanical ventilation during ICU admission for ≥ 48 hours during the study period, was included in the analysis. No other exclusion criteria were used. Extubation failure was defined as requiring endotracheal reintubation within 72 hours of prior extubation [1]. A subset of 100 randomly selected patients was used for derivation. The automated search algorithm was further validated against another set of 100 randomly selected patients from the cohort, with use of statistical software (Epi-info, Centers for Disease Control and Prevention, Atlanta, Georgia, [8]). The selection of 100 patients in both subsets was chosen to limit manual annotation burden while ensuring a robust sample size for the two subsets.

### Manual data extraction strategies

No implicit gold standard is available for the identification of extubation failure, but manual data extraction is the traditional method for assessing data in clinical research. The medical records of the derivation and validation groups were reviewed manually and independently by two critical care clinicians (M.R. and S.H.). Data was collected from procedure notes marked as *critical care progress note, respiratory care note*, *intubation, chest x-ray* and flow sheet marked as *respiratory care* and then reviewed to assess the presence and timing of extubation failure. The accuracy or timing, or both, of reintubation using notes and flow sheets have not been validated previously. However, there is no other form of documentation regarding the ICU intubation in patient medical records. At our institution, *intubation* notes are required in documentation of reintubation in the ICU. Additional factors that represent reintubation, such as mechanical ventilation parameters, were not used because these may be present for patients receiving noninvasive ventilation, as well as patients who require ventilatory support through a tracheotomy. The research team performing manual data extraction was not aware of the automated electronic note search strategy results.

### Automated electronic search strategy

The present retrospective study used data from the Mayo Clinic Life Sciences System [9], an exhaustive database of patient information which has been validated previously and is reliable [10,11]. Centralized for all Mayo Clinic hospital data, this database contains such patient information as demographic characteristics, diagnoses, laboratory test results, flow sheet data, and clinical and pathologic information gathered from various resources in the institution. We used a Web-based commercial software tool set (Data Discovery and Query Builder [DDQB]; International Business Machines Corp) for data access. Within DDQB, a medical record can be searched for diagnosis codes (free text terms), laboratory test results, procedure codes, flow sheet row data, and other electronic medical record data. DDQB is based on Boolean logic to create free text searches [11]. With it, a researcher can search quickly for a unique entity by using a text search strategy.

Extubation failure data was extracted through a query that accessed flow sheet data using DDQB, returning the flow sheet row data equal to “Airway Tube Status” where patient’s nurses chart intubation/extubation status of the patient. Extubation failure was initially identified when term “extubation/extubated” was followed by term “intubation/intubated/inserted” within the defined period of 72 hours. The electronic search strategy was refined continuously through the addition or edit of terms to enhance sensitivity and specificity to greater than 90% in the derivation subset. The performance improved, when the flow sheet row data equal to “Airway Tube Type” where the patient’s nurses chart presence of endotracheal tube/tracheostomy/nasotracheal tube was added to the search query. The final search used to build the automated electronic search identified extubation failure if term “extubation/extubated” was followed by term “intubation/intubated/inserted” within the defined period of 72 hours and when “Airway Tube Type” was “endotracheal tube”.

Restrictions were placed on location of endotracheal intubation whereby the included intubations were only those performed during the time period of each specific ICU admission where an extubation had occurred previously in the same ICU admission, in the ICU, as opposed to those intubations that were performed in the emergency department or in the operation room.

To validate the automated electronic search, sensitivity and specificity were calculated through comparison to the reference standard of comprehensive manual medical record review (Figure 1). The automatic search was done by an independent critical care researcher (G.W.).

### *Ethics declaration*

The study was conducted in accordance with the Declaration of Helsinki. The study was approved by the Mayo Clinic Institutional Review Board (IRB) for the use of existing medical records of patients who gave prior research authorization. The IRB conducted a risk-benefit analysis, and determined the study constitutes minimal risk research. The IRB also approved waiver of the requirement to obtain informed consent in accordance with its policy as justified by the investigators, and waiver of HIPAA authorization in accordance with applicable HIPAA regulations.

### *Statistical analyses*

We calculated sensitivity and specificity of the automated electronic note search strategy on the basis of comparisons of test results and the reference standard in both the derivation and validation patient subsets using online clinical calculator (http://www.vassarstats.net/clin1.html). The 95% confidence intervals were calculated with an exact test for proportions. We used statistical software Epi-Info (Centers for Disease Control and Prevention, Atlanta, Georgia) for all other data analysis.

## Results

In the derivation subset, 14 of the 100 patients had reintubation performed within 72 hours of their prior endotracheal extubation. The automated electronic search strategy achieved a sensitivity of 85% (95% CI, 56%-97%) and a specificity of 95% (95% CI, 87%-98%) in the derivation subset (Table 1). Disagreement between the automated electronic search strategy and the manual review occurred in 6 patients in this data subset. On review of these 6 medical charts, we noted that four of these patients identified by electronic search as extubation failure had “nasal trumpet” listed as “Airway Tube Type” after initial extubation and “Airway Tube Status” was listed as “inserted”. On the basis of this finding, the automated electronic note search strategy was refined (see Table 1) to arrive at our final automated search algorithm in which the sensitivity was 93% (95% CI, 64%-99%) and specificity increased to 100% (95% CI, 95%-100%) in the same derivation subset of 100 patients. In the validation subset of 100 randomly selected patients, the automated electronic search strategy achieved a sensitivity and specificity were of 94% (95% CI, 69%-99%) and 98% (95% CI, 92%-99%) respectively. Disagreement between the automated electronic search strategy and the manual review occurred in 5 patients (4 false positives and 1 false negative). On review of these 5 medical charts, we noted that four of these patients were identified by electronic search as extubation failure (false positives). Two patients had accidental extubation and prompt reintubation, while two had re-intubations briefly (less than 2 hours in both cases) for procedures and re-extubation shortly thereafter. These cases were not identified or missed as extubation failure by manual search. One patient was not identified as extubation failure by the electronic strategy (false negative) because “Airway Tube Status” did not list “extubated” at the initial extubation.

**Table 1:** Derivation and validation of electronic note search algorithm

	**Search algorithm**	**Sensitivity% (95% CI)**	**Specificity% (95% CI)**
Initial signature	Airway Tube Status: Intubated/Inserted within 72 h of being extubation/extubated	85% (56%-97%)	95% (87%-98%)
Final signature	Airway Tube Status: Intubated/Inserted within 72 h of being extubation/extubated	93% (64%-99%)	100% (95%-100%)
	Airway Tube Type: Endotracheal tube		
Validation	Airway Tube Status: Intubated/Inserted within 72 h of being extubation/extubated	94% (69%-99%)	98% (92%-99%)
	Airway Tube Type: Endotracheal tube		

The initial negative predictive value (NPV) and positive predictive value (PPV) in the derivation subset was 97% (95% CI, 90%-99%) and 75% (95% CI, 47%-91%) respectively, with a prevalence of 0.14. Posttest refinements were made within the same derivation subset. In the validation subset, the automated electronic search strategy achieved a concordance for NPV of 98% (95% CI, 92%-99%) and for PPV of 94% (95% CI, 69%-99%).

## Discussion

The results of the present study indicate that the development and validation of an electronic search algorithm within the EMR for identifying patients who have extubation failure in the ICU is a reliable alternative to a manual chart review. By using this electronic search algorithm, we achieved both sensitivity and specificity greater than 90%, with an NPV of 98% and a PPV of 94%. This study’s findings are in accordance with previously published studies showing that the use of electronic search strategies offers highly valid and reliable data extraction methods [11].

Disagreement between our electronic note search strategy and manual medical record review occurred in 6 cases in the derivation subset. We reviewed these 6 charts and the reasons for the misclassifications. The top reason, which accounted for 66% of misclassifications, related to the “Airway Tube Type” inserted not being endotracheal tube.

This process was a necessary first step in identifying extubation failure in the critical care setting before evaluating the risk factors that may be associated with extubation failure. With EMR adoption, a vast amount of information can be assimilated quickly. If these records were analyzed retrospectively, time constraints may become a barrier because of the abundance of information.

The use of electronic search strategies has been increasing in the past decade with the increase in EMR adoption and the ability to combine distributed data sources [6,7,11]. Survey findings from the Centers for Disease Control and Prevention reported an increase in EMR use by US office-based physicians from 18% in 2001 to more than 50% in 2011 [12]. The push to adopt the EMR has been driven in large part by the US government. Incentive programs developed by the federal government promote adoption of EMRs, including the Health Information Technology for Economic and Clinical Health Act in 2009 [13,14].

However, with the adoption of EMRs, the amount of information that can be assimilated is enormous and can potentially lead to barriers in clinical research. Development of electronic search algorithms can prove useful for clinical and research purposes. Thus, to identify risk factors associated with extubation failure, it is necessary to first identify whether extubation failure took place. After this is accomplished, identification of the risk factors resulting in this end point can be performed. Similar search algorithms may be derived and validated to identify other outcomes or events of interest. In ICU setting, these may include patient comorbidities [6], emergent intubation [7], use of noninvasive ventilation before intubation and after extubation etc.

Several studies address the complications related to extubation failure in the ICU [15,16]. However, these investigations are mainly prospective studies with small sample sizes and therefore the information is easier to acquire. For example, to obtain the described data from manual medical record review, the time invested by our research team ranged from five minutes to more than ten minutes per medical record. The automatic electronic note search strategy was derived from DDQB using keyword phrases within electronic flow sheet. This approach resulted in a tremendous reduction in time commitment compared with manual medical record review. Furthermore, the strategy not only is useful for research purposes, but also is of value in the patient care setting [14]. For example, the strategy may be used for identification of patients with extubation failure in “near real time” allowing possible interventions such as automated reinitiation of ventilator bundle or enrollment in clinical trials. The search strategy distinctly differs from the recently advanced approach of “natural language processing” in the following ways. We performed a free text search for a limited number of keywords. Essentially, this was a simple search to match words. Natural language processing is semantic mapping of uncertain text to controlled terminology (Systematized Nomenclature of Medicine-Clinical Terms [SNOMED CT], [17]). Natural language processing requires dedicated real-time software and hardware that may make the system more complex and less reliable. A direct query was submitted to a database using standard open database connectivity connection. Because DDQB is commercial-based software and the medical record is transitioning from paper to electronic, our approach is applicable to any electronic health system.

The search strategy used in this context has several limitations. First, performance of the electronic note search strategy is dependent on the foundation of information from which it is derived. Inconsistencies in the database and text search phrases can lead to inaccurate results and thus limit the applicability of this approach to areas with a similar database. For example, although we used a free text search, we performed this task within a structured flow sheet. The electronic flow sheet has a limited number of designations in the fields we searched, which increases the specificity score (i.e., “Intubation/inserted vs extubation”). Therefore, the search algorithm used may be aided by a natural language processing approach to algorithm development. Second, our electronic search focused only on electronic flow sheet within the critical care setting. It might be possible that if all the clinical notes were analyzed during the specified time interval, our results may have been different. This analysis was not feasible because of the time barrier involved in reviewing the entire medical record during the specified interval. Third, the timing of using any electronic search strategy is limited. Search algorithms are not real-time acquisition tools and depend on when the flow sheet data is posted. Therefore, they cannot be used in real time. Fourth, the iterative nature of query development requires independent validation of each modification. However, after the new query algorithm is built with optimal results, it could be automated. Fifth, data could have been entered in error or the database could have been corrupted [18]. However, this fifth limitation likely accounts for only a small proportion of the database. Sixth, data collection may be reported inaccurately, as with any retrospective study. Seventh, this comprehensive EMR system is unique to Mayo Clinic. However, the way in which the search strategy was performed can be applied to any standard or customized EMR software.

## Conclusions

Extubation failure within the critical care setting can be identified correctly with high sensitivity and specificity through the use of an automated electronic search algorithm. The achieved sensitivity and specificity can approach 100% through refinements in the electronic note search strategy and can serve to expedite clinical research and, ultimately improve, patient care. The present study reports on the development and validation of an electronic search algorithm regarding extubation failure in the ICU. Electronic search algorithms can subsequently be applied to ultimately determine risk factors associated with extubation failure in specific populations within critical care.

## Abbreviations

DDQB: Data discovery and query builder; EMR: Electronic medical record; NICU: Neuroscience intensive care unit; NPV: Negative predictive value; PPV: Positive predictive value; SNOMED-CT: Systematized nomenclature of medicine-clinical terms.

## Competing interests

All authors declare that they have no competing interests.

## Authors’ contributions

All authors contributed to the conception and design of the study, acquisition and interpretation of the data and the initial drafting and final revision of the manuscript for important intellectual content. All authors read and approved the final manuscript.

## Pre-publication history

The pre-publication history for this paper can be accessed here:

http://www.biomedcentral.com/1471-2253/14/41/prepub
